# Supplementary material for: Suppressed Phase Separation of Mixed-Halide Perovskite Quantum Dots Confined in Mesoporous Metal Organic Frameworks
Source: Nanomaterials (Basel). 2023 May 16;13(10):1655. doi: 10.3390/nano13101655 (PMC10222501; doi:10.3390/nano13101655)
Supplement: Supplementary file 1 [file nanomaterials-13-01655-s001.zip › nanomaterials-2294946-supplementary.pdf]

Supporting Information

Suppressed phase separation of mixed-halide perovskites quantum dots confined in mesoporous metal organic frameworks

Duanqi Ma[1†], Yanlin Xu[1†], Qiuying Chen[1], Huafeng Ding[1], Xiaoming Tan[1*], Qinfeng Xu[1] and Chuanlu Yang[1]

[1]Department of Physics and Optoelectronic Engineering, Ludong University, Yantai 264025, China
†These authors contribute equally to this work.
* Correspondence: author: mingtanxiao@ldu.edu.cn

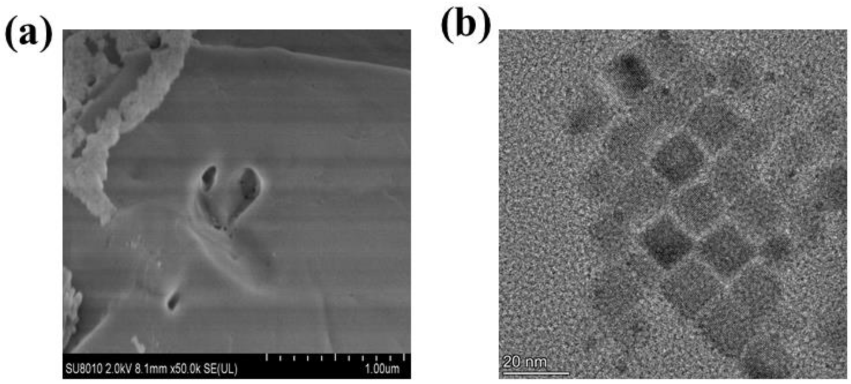

Figure S1. (a)The SEM of MOF-5, (b) The TEM of $CsPbBr_{1.5}I_{1.5}$.

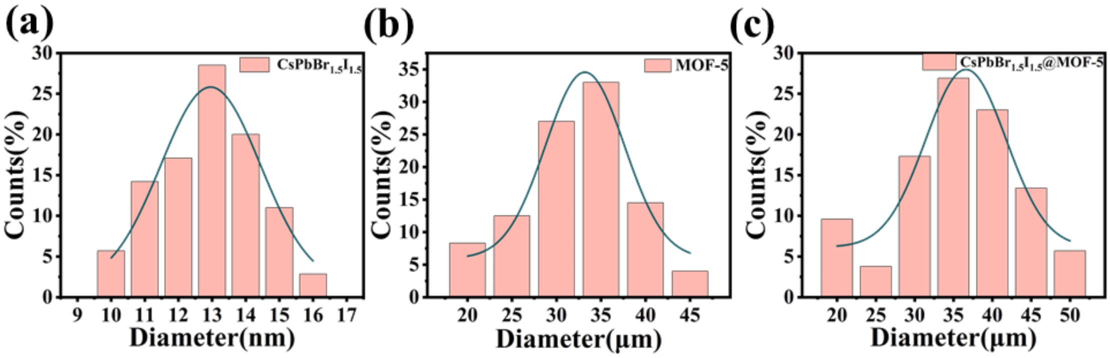

Figure S2. The size statistics of (a)$CsPbBr_{1.5}I_{1.5}$, (b)MOF-5 and (c)$CsPbBr_{1.5}I_{1.5}$/MOF-5.

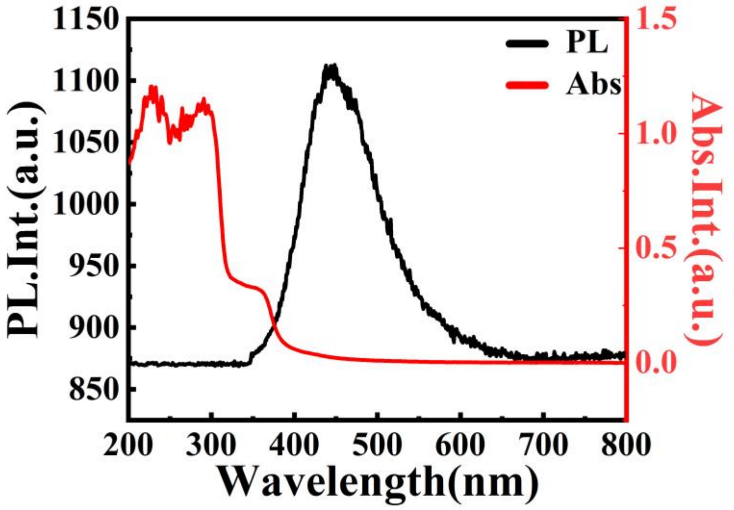

Figure S3. The PL and absorption spectrum of pure MOF-5.

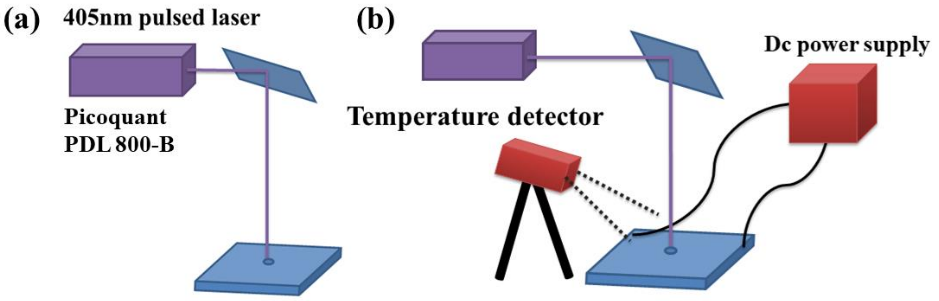

Figure S4. (a) The schematic diagram of laser irradiation experiment, (b) The schematic diagram of phase separation experiment with heat effect.

Fig. S4a shows the process of laser irradiation, 405nm pulsed laser was directly irradiated on the sample, and the excitation light of the sample was collected and measured by CCD. Fig. S4b is the process of heating, a DC power supply is used to provide an electric current to the conductive glass sheet, changing the temperature of the sample base by varying the magnitude of the current. At the same time, a temperature detector was placed to detect the substrate temperature in real time. The experimental light path is the same as the laser irradiation path, and the luminescence of the sample is collected by CCD.

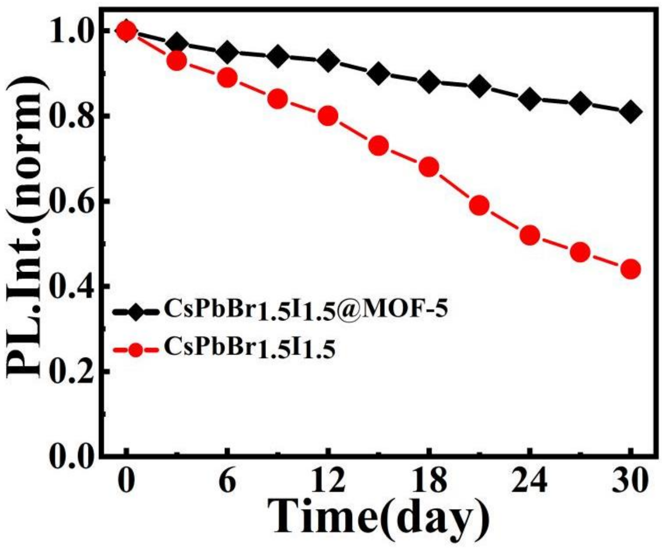

Figure S5. The stability of $CsPbBr_{1.5}I_{1.5}$/MOF-5 and $CsPbBr_{1.5}I_{1.5}$ with time.

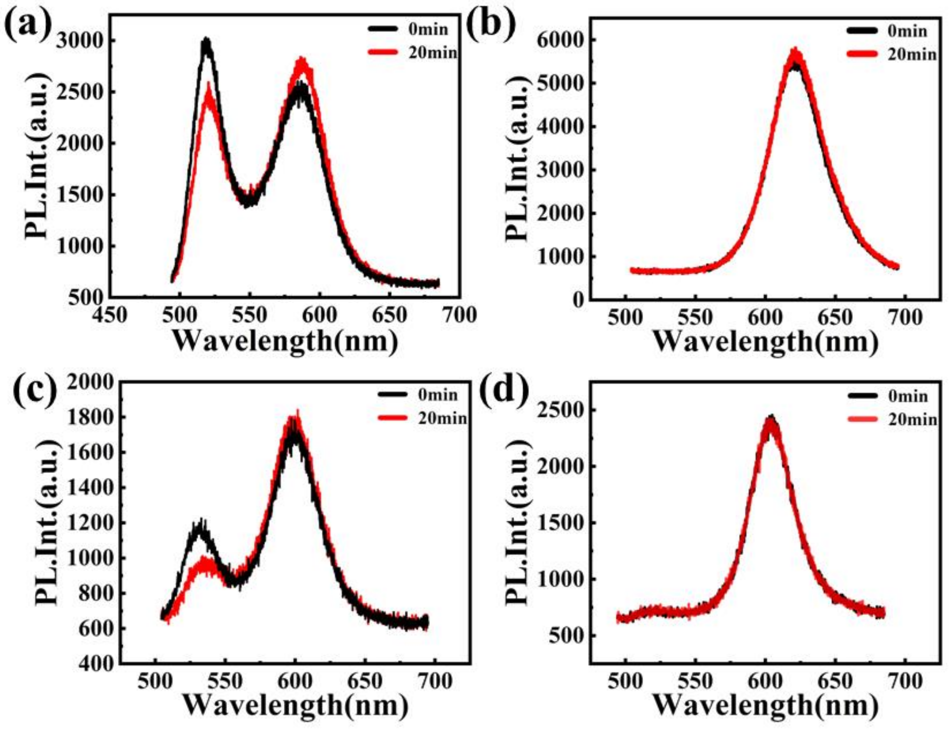

Figure S6. (a)The PL change of $CsPbBr_{1.5}I_{1.5}$ in 20min after 20μW laser irradiation, (b)The PL change of $CsPbBr_{1.5}I_{1.5}$/MOF-5 in 20min after 20μW laser irradiation, (c) After heating at 60°C for 3min, $CsPbBr_{1.5}I_{1.5}$ PL changes within 20min, (d) After heating at 60°C for 3min, $CsPbBr_{1.5}I_{1.5}$/MOF-5 PL changes within 20min.
